# Rescue of virulent class I Newcastle disease virus variant 9a5b-D5C1

**DOI:** 10.1186/1743-422X-9-120

**Published:** 2012-06-18

**Authors:** Yang Yu, Xusheng Qiu, Dan Xu, Yuan Zhan, Chunchun Meng, Nana Wei, Hongjun Chen, Lei Tan, Shengqing Yu, Xiufan Liu, Aijian Qin, Chan Ding

**Affiliations:** 1 College of Veterinary Medicine, Yangzhou University, 48 Wenhuidong Road, Yangzhou, 225009, People’s Republic of China; 2 Shanghai Veterinary Research Institute, Chinese Academy of Agricultural Sciences, Shanghai, 200241, People’s Republic of China; 3 China National Engineering Technology Research Centre for Poultry, 2949 Zhennan Road, Shanghai, 200331, People’s Republic of China

**Keywords:** Newcastle disease virus, Reverse genetics, Minigenome, Helper plasmids

## Abstract

**Background:**

The virulent class I Newcastle disease virus (NDV) variant 9a5b was generated from a nonvirulent NDV isolate Goose/Alaska/415/91 via nine consecutive passages in the chicken air sac, followed by five passages in the chick brain. The evolutionary mechanism of virulence in the class I NDV isolate is not fully understood. To elucidate this evolutionary mechanism, a reverse genetics manipulation specific for class I NDV is indispensable.

**Results:**

A full-length cDNA clone of 9a5b and the helper plasmids pCI-NP, pCI-P, and pCI-L were constructed from segments of cDNA. After these plasmids were co-transfected into BSR T7/5 cells, infectious viral particles were obtained. The rescued viruses were genetically and biologically identical to the parental strain and showed similar pathogenicity in chickens.

**Conclusion:**

A stable recovery method for class I NDV was established. Reverse genetics of the class I NDV variant 9a5b allowed for the generation of genetically altered and virulent NDV, and can be used as a foundation for research on the evolution of virulence in class I NDV isolates.

## Introduction

Newcastle disease (ND) is one of the most serious and lethal avian diseases, resulting in heavy losses within the poultry industry worldwide [1-3]. Its causative agent, virulent Newcastle disease virus (NDV), is classified as the Avulavirus genus within the paramyxovirinae subfamily of the paramyxoviridae [4,5]. The genome of the virus is composed of a single-stranded, negative-sense RNA approximately 15 kb in length that encodes six structural viral proteins: nucleoprotein (NP), phosphoprotein (P), matrix protein (M), fusion protein (F), hemagglutinin-neuraminidase (HN) and the large protein (L) [6]. In addition, two non-structural proteins, V and W, are also encoded by the P gene of NDV via the RNA editing mechanism [7,8].

NDV isolates were classified into two major categories class I and class II. Class II NDV includes nine well-established genotypes labeled by Roman numerals. Class I NDV was identified approximately 6 years ago and has since proved to be wide-spread in avian species [9,10]. Class II genotypes have been well researched, but the significance of class I isolates is not well understood [11].

Class I NDV has several different poultry hosts, most of which do not experience any observable symptoms of viral infection [12-14]. Although class I virus isolates are not usually virulent, evolution of virulence in the class I strains has been reported. Yu et al passaged the nonpathogenic class I NDV isolate of Goose/Alaska/415/91 nine times through the specific pathogen free (SPF) chicken air sac and, subsequently, five times through the chicken brain [15]. Virulence gradually evolved in the viral isolate; the final generation of the virus was designated 9a5b and it displayed virulence equivalent to that of the typical velogenic NDV strain, Herts/33. Few differences in nucleotides were found between the genomes of these viruses. The F motif had mutated from that in ERQER/L (as in the parent strain, Goose/Alaska/415/91) to resemble the typical virulent type, KRQKR/F; the HN length was changed from 616 aa to 572 aa; however, no detectable change in the virulence was observed in Goose/Alaska/415/91 when it was passaged more than 15 times through embryonated eggs [15,16]. This research has raised several issues that need to be addressed including the relevance of the cellular materials in the chicken air sac compared to that of eggs in the development of pathogenic NDV, and the potential causal or functional relationship between the mutations in HN and F. In our research, we wanted to determine the mechanisms involved in the process of NDV virulence evolution. Since there is little information available, a reverse genetics manipulation for NDV strain 9a5b-D5C1 was constructed as a foundation to assess NDV virulence evolution.

## Results

### Construction of a full-length cDNA clone from the 9a5b-D5C1 genome

To avoid interference from quasispecies, the original 9a5b strain was plaque-purified by five passages through DF1 cells. During the plaque purification, the biggest plaque from each generation of passage was collected and used for the subsequent passage. The resulting clone 9a5b-D5C1 (designate as D5C1 thereafter) was utilized for the subsequent recovery. Fragments of cDNA clones spanning the entire viral genome were transcribed from viral RNA with 6-nt random primers and then amplified by polymerase chain reaction (PCR) for sequencing. To ensure precision, the genome of D5C1 was sequenced at least five times. The full length genomic sequence of D5C1 was compiled and submitted to the GenBank (accession number: JQ713944). D5C1 and the original 9a5b genomic sequence (accession number: AB524406.1) had greater than 99.99 % nucleotide identity. Only six nucleotides were found to differ from the consensus reported in Japan: 3628 C(T), 7406A(T), 9511 C(T), 9513 C(T), 9514 C(T), and 9988 G(A) (consensus sequence in parentheses).

**Figure 1:**
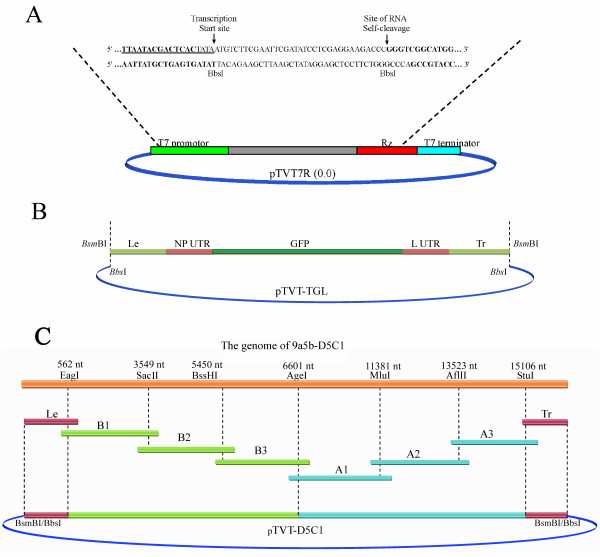
**Schematic representation of the minigenome pTVT-TGL and construction of the full-length cDNA plasmid pTVT-D5C1.** (**A**) The reverse genetics vector pTVT7R(0.0); (**B**) the minigenome pTVT-TGL; (**C**) the full-length cDNA plasmid pTVT-D5C1. To contruct full-length cDNA plasmid of pTVT-D5C1, eight PCR fragments spanning the following positions were amplified: Le, nt 1 to nt 562 (*Eag*I); B1, nt 562 (*Eag*I) to nt 3549 (*Sac*II); B2, nt 3549 (*Sac*II) to nt 5450 (*Bss*HI); B3, nt 5450 (*Bss*HI) to nt 6601 (*Age*I); A1, nt 6601 (*Age*I) to nt 11381 (*Mlu*I); A2, nt 11381 (*Mlu*I) to nt 13523 (*Afl*II); A3, nt 13523 (*Afl*II) to nt 15106 (*Stu*I); and Tr, nt 15106 (*Stu*I) to nt 15198. All the fragments were orderly joined with pTVT7R(0.0). The segment Le followed the T7 promoter; the segment Tr was followed by the hepatitis delta virus (HDV) ribozyme and the T7 terminator. For pTVT-TGL, the fragment was inserted into pTVT7R(0.0) in the reverse direction.

Based on verified sequences of D5C1, a NDV full-length cDNA clone was assembled from seven segments of cDNA and subcloned into vector pTVT7R(0.0) (Figure 1A). In the resulting plasmid, TVT-D5C1, the full-length NDV cDNA was flanked with the T7 RNA promoter and the hepatitis delta virus (HDV) ribozyme motif.

### Verification of helper plasmids

The co-expression of viral nucleoprotein (NP), phosphoprotein (P) and large protein (L) is essential for recovery of paramyxoviruses. By using the full-length cDNA clone as the template, the complete coding region of the NP, P and L genes was obtained using PCR. The NP and P gene were amplified by high-fidelity RT-PCR and ligated into the pCI neo vector (Promega, Madison, WI, USA) to obtain the plasmids pCI-NP and pCI-P. Three overlapping PCR products L1, L2 and L3, were amplified and cloned sequentially into the pCI neo vector to obtain pCI-L. The sequence consistency of the three helper plasmids was verified by sequencing at Sangon Biotechnology (Shanghai, China).

To determine the usefulness of helper plasmids, a minigenome plasmid was prepared as well. In this plasmid, the green fluorescence protein (GFP) gene was flanked by the leader (Le), 5' untranslated region (UTR) of NP and 3' UTR of L, the trailer (Tr) (Figure 1B). The Le and Tr were involved in assembly of mini-genomic RNA by NP protein. 5' UTR of NP contains a transcription start component for viral RNA polymerase, while 3' UTR of L contains a transcription termination component. The minigenome fragment was inserted into vector pTVT7R(0.0) to obtain plasmid TVT-TGL, which was verified by sequencing.

**Figure 2:**
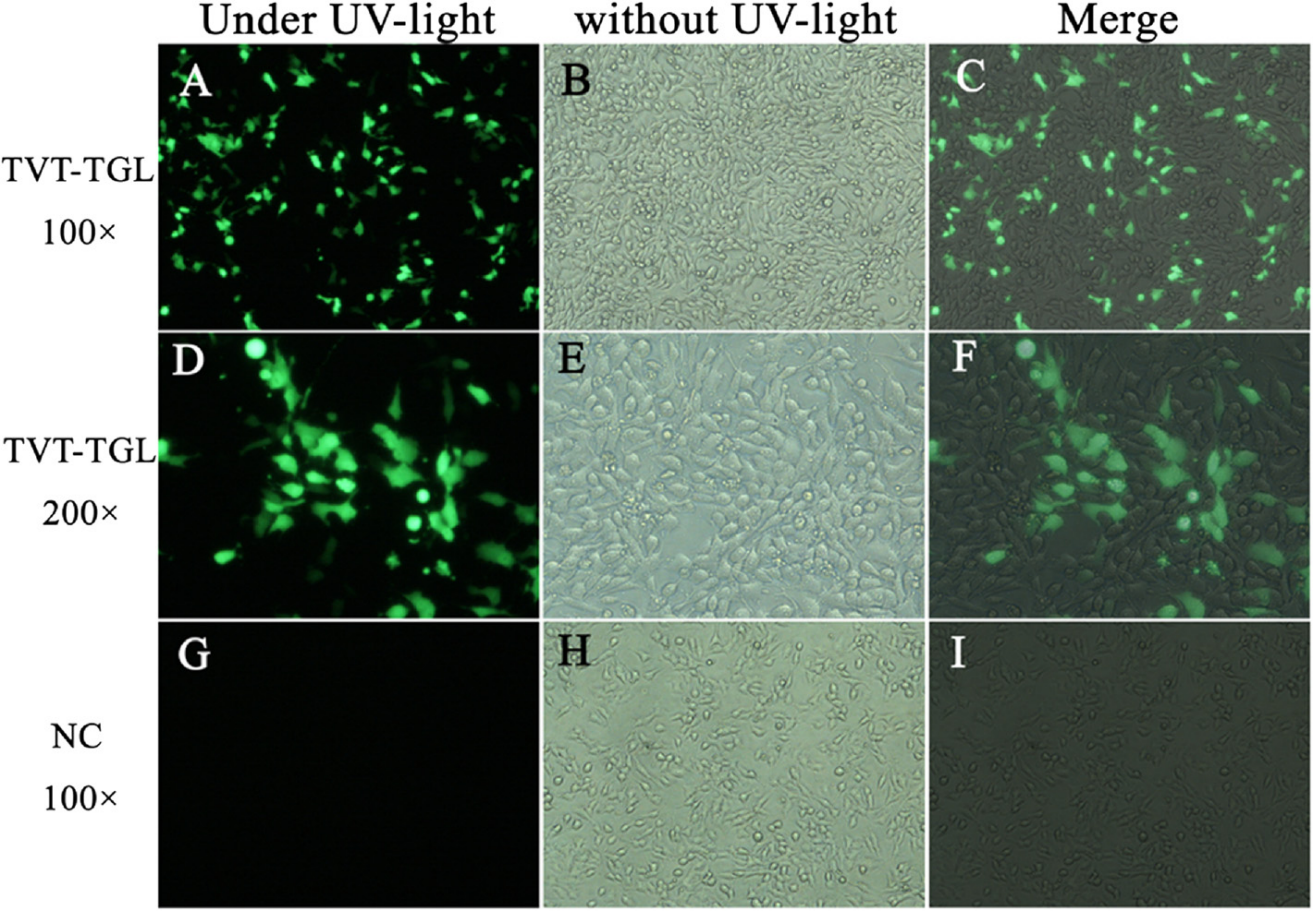
**Verification of the helper plasmids pCI-NP, pCI-P and pCI-L in BSR-T7/5 cells co-transfected with minigenome pTVT-TGL.** Green fluorescence was observed under fluorescence microscopy in minigenome-transfected cells 48 h post-transfection. Panel “TVT-TGL 100×”: Cells transfected with pCI-NP, pCI-P, pCI-L and pTVT-TGL (100×); Panel “TVT-TGL 200×”: Cells transfected with pCI-NP, pCI-P, pCI-L and pTVT-D5C1 (200×); Panel “NC 100×”: Cells transfected with pTVT-TGL (100×) only. UV-light: ultraviolet.

To examine the efficiency of the helper plasmids pCI-NP, pCI-P and pCI-L, BSR-T7/5 cells were co-transfected with pCI-NP, pCI-P, pCI-L and pTVT-TGL. In BSR-T7/5 cells, the T7 RNA polymerase will transcribe single-strand (−) RNA from pTVT-TGL. If the helper plasmids were functional, those plasmid-coded viral proteins will assemble into viral RNA-dependent RNA polymerase (vRdRp) to transcribe single-strand (+) RNA as well as mRNA of GFP from (−) RNA. At 48 h post-transfection, green fluorescence was observed in cells which were transfected with the minigenome pTVT-TGL and helper plasmids. In contrast, there was no visible green fluorescence in those cells in which only TVT-TGL was transfected (Figure 2). This result suggested that the helper plasmids can be successfully used for recovery.

### Recovery of infectious recombinant NDV from cDNA

The full-length cDNA clone, pCI-NP, pCI-P and pCI-L were co-transfected into BSR T7/5, in which the intracellularly-expressed T7 RNA polymerase generated the first strand of viral genomic RNA. Then, vRdRp assembled from NP, P and L was expressed in cells, and the vRdRp participated in the duplication of more viral genomic RNA strands. Two parallel experiments were performed as the controls: the minigenome plasmid TVT-TGL was substituted for the cDNA clone, and pCI-L was removed from the superfection mixture.

After 24 h, the cells transfected with the minigenome started to glow under UV light, suggesting that transfection had been successful. All three kinds of cells were serially passaged (up to three times) in SPF embryonic eggs. The hemagglutination (HA) titers of rescued NDV in the three passages were 2^7^, 2^8^, and 2^7^ respectively. As expected, both parallel control experiments displayed no hemagglutination, no matter how many passages were performed.

In order to identify the viruses, rescued viral RNA was extracted from fresh allantoic fluid, and the full-length genome was sequenced as described above. It was found that the genomic sequence of the descendant virus was identical to that of the parental virus, with only one exception: C at nt 14,227 instead of the original T. This unexpected mutation was located in the open reading frame of the L protein sequence and introduced no amino acid change; the mutation could be considered a genetic marker of rescued virus particles.

### Biological characterization of the generated virus

Three standard pathogenicity tests of mean death time (MDT), intracerebral pathogenicity index (ICPI) and the intravenous pathogenicity index (IVPI), were performed to determine the differences in virulence between rescued D5C1 and the original virus. The MDT value of the rescued virus was 54, slightly higher than that of its parental strain. In the ICPI test, all the ten chicks died, and they had an ICPI value of 1.89. The rescued D5C1 and the original virus also displayed similar values of 2.7 and 2.8, respectively, in the IVPI test. Chickens in both challenge groups were sick 36 h post-challenge, and all were dead 72 h post-challenge. Taken together, these data show that rescued NDV has a similar pathogenicity as its parental virus.

**Figure 3:**
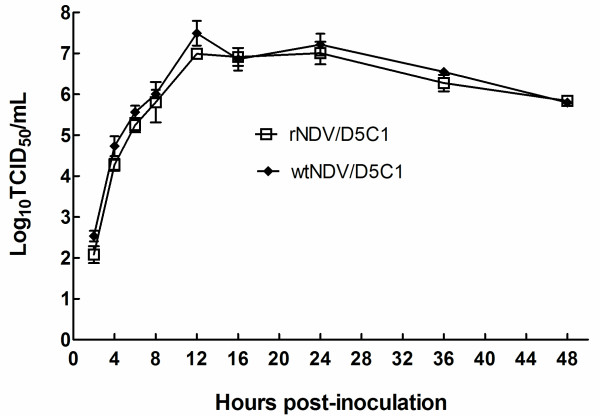
**Growth curves of wtNDV/9a5b-D5C1 and rNDV/9a5b-D5C1 in DF1 cells.** Cell monolayers with 80 % confluence in a 6-well plate were transfected with wtNDV/9a5b-D5C1 and rNDV/9a5b-D5C1 at a multiplicity of infection (MOI) of 0.01, and the supernatant was harvested at 2, 4, 6, 8, 12, 16, 24, 36, and 48 h post-infection. The 50 % tissue culture infection dose (TCID_50_) of the virus was determined to quantify the live viruses secreted into the supernatant at each time point. All titrations were performed in triplicate. Bars show standard deviations.

To compare the propagation capability of these viruses, growth curves were constructed in the DF1 cells for both the parent and the descendent virus. As shown in Figure 3, a similar TCID_50_ was found at all the time points. This result revealed that the kinetics and rate of replication of rescued NDV are comparable to those of its strain of origin.

## Discussion

Recently, several recombinant Newcastle disease viruses (rNDV) have been generated by genetic techniques [17-23]. This manipulation has facilitated the study of the molecular mechanisms behind the virulence, pathogenicity and evolution of NDV. To date, all NDV strains isolated worldwide have been classified into two major categories: class I and class II [10,24-26]. Class II contains the well-researched NDV strain genotypes, while class I viruses were only identified in 2006 and have been studied less intensely [10]. Globally, class I NDVs are commonly isolated from domestic poultry and wild bird species [27-29]. Class I NDV is the dominant class, especially in developed countries where NDV is not endemic in poultry. It is thought that class I NDV interferes with the NDV vaccine [24]. Little is known about the evolution of class I NDV, as most NDV research has been performed on class II viruses.

Class I viruses are generally nonvirulent, except for two known virulent class I viruses [24,30,31]. There is, however, a concern that class I NDVs have the potential to evolve virulence. In 2002, Yu et al reported a virulent class I NDV variant that originated from a nonvirulent isolate Goose/Alaska/415/91 after nine consecutive passages by air-sac inoculation and five passages through chicken brain [15]. Sequence analysis showed that the fusion protein cleavage site was mutated from KRQKR/F of Goose/Alaska/415/91 to ERQER/L, a typical motif of virulent NDV, in 9a5b [32].

To determine the molecular mechanism behind class I virus evolution, reverse genetics techniques specific for class I NDV are useful; however, all the previous rescued NDVs belongs to class II, and prior to our research, there was no recovery-generated recombinant class I NDV.

In this study, the first virulent recombinant class I NDV variant was generated from the full-length cNDV clone. First, the entire genomic viral cDNA was ligated into the vector pTVT7R(0.0). However, the plasmids containing viral cDNA greater than 12 kb in length propagated inefficiently in host bacteria cells. Different competent cells, media and plasmid extraction methods did not increase the yield of those plasmids, which maintained a concentration of 50–100 ng/μl (OD_260/280_ ranging from 1.8 - 1.9). These results suggested that the genomic cDNA from the class I NDV strain was unstable in the bacterial carrier.

In order to obtain more favorable results, the recovery procedure was modified. All of the plasmids containing a long viral cDNA fragment were identified using a set of specific primer pairs for every 1 kb of the full-length genome (due to the large number of primers, the data are not shown). Only the positive plasmids were re-transformed into competent cells and extracted with QIAGEN Plasmid Midi Kits for the following experiments.

In spite of the low yield of the full-length cDNA clone, the reverse genetics transfection system used in this study proved to be efficient. The full-length plasmid (50 ng/μl) was utilized in the rescue manipulation, and rescued viruses were detected in all three trials containing 20 μl, 25 μl or 30 μl of full-length plasmid respectively, suggesting that the plasmid was sufficient to rescue NDV. We repeated the work and obtained similar results.

In this recovery manipulation, the first strand of viral genome-like RNA was transcribed from the full-length clone by T7 RNA polymerase in the cytoplasm of BSR T7/5. Then, the HDV ribozyme located in the 3’ end of the transcribed RNA cut itself out together with the un-required RNA fragment. In this way, a negative strand RNA with no methylation at its 5’ end and no polyadenylation at its 3’ end was generated, resembling the genomic (−) RNA of NDV. Meanwhile, vRdRp of 9a5b was assembled from the NP, P and L proteins encoded by co-transfected helper plasmids. Using (−) viral RNA as a template, the vRdRp generated (+) viral RNA, together with all eight strands of viral mRNAs, which encoded the viral proteins required for the generation of infectious viral particles.

## Conclusion

In this study, we constructed a full-length cDNA clone and the helper plasmids pCI-NP, pCI-P, and pCI-L from cDNA of 9a5b-D5C1. After these plasmids were co-transfected into BSR T7/5 cells, infectious viral particles were obtained. The rescued viruses were genetically and biologically identical to the parental strain and showed similar pathogenicity in chickens, suggesting that a stable recovery manipulation for class I NDV had been established. The reverse genetics manipulation of 9a5b developed here allows the generation of genetically altered NDV, which can be used for research on the evolution of class I NDV isolates.

## Methods

### Virus, plasmids and cells

The virulent NDV strain 9a5b was obtained from Professors Toshihiro Ito and Koichi Otsuki in Tottori University, Japan [31]. The virus was plaque-purified five times in DF1 cells, and the final pure clone used in this study was named D5C1 and was grown for 9–11 days in SPF embryonated eggs. BSR T7/5 cells were obtained from Dr. Zhigao Bu in Harbin Veterinary Research Institute, Chinese Academy of Agricultural Sciences, China. The cells were developed by Buchholz et al [33] and they express the phage T7 RNA polymerase in a stable manner in culture. The cells were maintained in DMEM (Gibco, USA) supplemented with 10 % fetal calf serum (FCS) and 1 mg/ml G418 (Sigma, Saint Louis, MO, USA), as previously reported [20,33,34]. The vector pTVT7R(0,0) was a generous gift from Andrew Ball [20,34]. In this vector, the T7 promoter sequence and the hepatitis delta virus (HDV) ribozyme sequence flank the insertion site for the full-length NDV cDNA (Figure 1A). After it was transcribed from the plasmid with T7 polymerase in the BSR T7/5 cells, the resulting RNA is an exact copy of the parental NDV genome [34,35].

### Cloning and assembly of the full-length NDV cDNA clone

Fresh allantoic fluid from the D5C1 virus was directly utilized for the isolation of viral genomic RNA using the Trizol RNA extraction kit (Invitrogen, Carlsbad, CA, USA). The cDNA was reverse transcribed from viral RNA with a 6-nt random primer or a specific primer (5'-ACC AAA CAG AGA ATC-3') complementary to the 3' end of the NDV genomic RNA.

In order to determine the exact full-length genomic sequence of the virus, nine successive and overlapping DNA fragments were amplified by PCR with a set of specific primer pairs for class I NDV. The 3' and 5' ends of the viral genome were obtained by T4 RNA ligase-mediated rapid amplification of the cDNA end (RLM-RACE). All of the above experiments were performed as reported previously [26]. RT-PCR products from overlapping fragments covering the full-length genome were extracted from the agarose gel, ligated into the TA cloning system (Promega, Madison, WI, USA) and transferred into *E. coli* DH5α. At least five clones of each segment were sent to Sangon Biotechnology (Shanghai, China) for sequencing. The sequences of overlapping DNA fragments were aligned and then compiled into a correct and precise consensus full-length genome sequence using the Lasergene software package (DNASTAR Inc. Madison, WI 53715, USA).

According to the restriction enzyme site maps, a set of primers was designed to amplify nine overlapping fragments (Table 1). Segment Le and Tr, flanked by the *Bsm*BI and *Spe*I restriction enzyme sites respectively, were digested and ligated with *Bbs*I-digested vector pTVT7R(0.0) to construct plasmid TVT-3'5'. Segment A, which was the ligation product of segments A1, A2 and A3, was digested by *Age*I and *Stu*I and then ligated into TVT-3'5' to obtain plasmid TVT-A. Segments B1, B2 and B3 were also inserted into TVT-3'5' in order to obtain TVT-B. Finally, a full-length cDNA clone, TVT-D5C1, was completed via ligation of *Age*I-*Sal*I double-digested TVT-A and TVT-B (Figure 1C).

**Table 1:** Primers used in this study to generate overlapping PCR fragments from the genome of D5C1

**Fragment designation**		**Primer sequence (5'-3')**	**Position**^c^	**Expected product size (bp)**
**Le**	F^a^	ACCAAACAGAGAATCCGTGAG	1-21	2280
	R^b^	TGGACGATTTATTGCTAAGCTTG	2258-2280	
**B1**	F	CAAGACTGGAGCAAGCAACT	2219-2238	2003
	R	GGAGAGGCATTTGCTATAGG	4202-4221	
**B2**	F	GGGCTCAGTGATGTGCTCG	4100-4118	1964
	R	ATATAGGTAATGAGAGCAGATGTG	6040-6063	
**B3**	F	AAATAATATGCGTGCCACCT	5434-5453	1661
	R	GAACGCAGAGTAGAAAAGAATA	7073-7094	
**A1**	F	CAAGAACACCTGAATTTTATCCCG	6686-6909	2260
	R	TTAGATGCCTTTGGACCTGTTTTA	8922-8945	
**A2**	F	TGGTTTCACTCAAAATGGTCC	8876-8896	1223
	R	ATCCCTTCTGCCATTACCTG	10079-10098	
**A3**	F	ACCCTTGAGTACCTAAGAGATGA	9965-9987	2116
	R	TGTCCCCATAAGCCCAGAT	12062-12080	
**Tr**	F	CTAGGAAGAGCCTTAATTTGAT	13350-13371	1838
	R	ACAAAGATTTGGTGAATGACA	15167-15187	

^a^ F represents forward primer; ^b^ R represents reverse primer; ^c^ Positions were based on the genomic sequence of NDV D5C1 (accession number: JQ713944).

### Construction of helper plasmids

The full-length cDNA clone was utilized as a template to amplify the NP, P, and L genes, which were cloned into the eukaryotic expression vector pCI neo (Promega, Madison, WI, USA) to generate three helper plasmids.

To clone the NP and P genes, two primer pairs of PNF (5'-GA CTC GAG ATG TCC TCC GTA TTC GA-3') and PNR (5'- T * TCT AGA * CTA GTA TCC CCA GTC GG -3'), and PPF (5'-TA * CTC GAG * AGC ATG GCT ACG TTC AC-3') and PPR (5'-G * ACG CGT * TTA TCC ATT CAG TGC AA-3') (*Xho*I, *Xba*I and *Mlu*I sites are in italics and underlined) were used for PCR with AccuPrime™ Taq DNA Polymerase High Fidelity (Invitrogen); the PCR products were double-digested and then inserted in the multiple-cloning site of pCI-neo (Promega, Madison, WI, USA).

To clone the entire L gene, three overlapping segments, L1, L2 and L3, were amplified with three primer pairs: PL1F (5'-CGC GCT AGC AGA CCA TGG CAG GCT CCG GGC CC-3') and PL1R (5'-GTG GTT GAT GGC ATG CAT GAG CGA ATA G-3'), PL2F (5'-AAT GAC AAC CCG CGG CGT GAA CTG CTA TC-3') and PL2R (5'-CAG CAC AAC ATG GCA GCG GA-3'), and PL3F (5'-GCA CGA GCT AGA CTT AAG CGC TCC AGC ACG-3') and PL3R (5'-TAA GGT CGA CAA TCA ACA GCA TTT ACC ACT A-3'). The segment L2 was digested and cloned into the vector pCI-neo between the *Mlu*I and *Nhe*I sites. *Nhe*I-digested segment L2 and double-digested segment L3 were subsequently ligated into the vector in order.

### Construction of a minigenome plasmid for NDV D5C1

NDV D5C1 with a reporter gene of GPF was constructed. Three primer pairs, described below, were designed to amplify three sub-fragments.

The first pair, primers MLF (5'-CGT CTC TAC CCA CCA AAC AGA GAA TCT GTA AGG TAC GAT ATA AG-3') and MLR (5'-ACC ATC CCG GGA CGC GTA GAA GGT ACT CTC GAG CTT GAG CTT C-3'), were used to amplify fragment MLe from plasmid TVT-3'5' constructed above. The PCR product of MLe contained the sequence of leader and 5' UTR of NP protein, located from 1 nt to 121 nt of the viral genome of D5C1. The *Bsm*BI site (underlined) was added in the 5' end of primer MLF.

The second pair, primers MGF (5'-TTC TAC GCG TCC CGG GAT GGT GAG CAA GGG CGA GGA GC TGT T-3') and MGR (5'-GTT ACA GGT ACC GAT ATC TTA CTT GTA CAG CTC GTC CAT GCC G-3'), were used to amplify the fragment GFP from plasmid pEGFP-N1 vector (CLONTECH Laboratories, Inc.).

The third pair, primers MTF (5'-AAG TAA GAT ATC GGT ACC TGT AAC TGT TGA TAA TAG TGG TAA ATG C-3') and primer MTR (5'-T CGT CTC GTA TA**G GG**A CCA AAC AAA GAT TTG GTG AAT ATC AG-3'), were used to amplify fragment MTr from plasmid TVT-3'5' constructed above. The PCR product of MTr contained the sequence of 3' UTR of L protein and leader, located from 15,008 nt to 15,198 nt in the viral genome of D5C1. The *Bsm*BI site (underlined) and three extra G residues (bold) were added in the 5' end of primer MTR.

All three sub-fragments were joined together with primers MTF and MTR by overlapping PCR. The fusion fragment was digested with *Bsm*BI and then ligated with *Bbs*I-digested pTVT7R(0.0) vector. The recombinant plasmid was sent to Sangon Biotechnology (Shanghai, China) for sequencing.

### Cotransfection of helper plasmids with TVT-D5C1 or TVT-TGL

High-quality plasmid DNA from TVT-D5C1 and three helper plasmids were obtained from purification using QIAGEN Plasmid Midi Kits. BSR T7/5 cells were grown to 60 % confluency in 35 mm diameter dishes and then transfected with a total of 3 μg of plasmids using Lipofectamine^TM^ 2000 Reagent (Invitrogen) according to the manufacturer’s instructions. To determine the activity of the helper plasmid, 1 μg of pCI-NP, 0.5 μg of pCI-P and 0.5 μg of pCI-L were mixed with 1 μg of minigenome plasmid TVT-TGL in the transfection. Then, in the rescue process, 1 μg of TVT-D5C1 was utilized instead of pTVT-TGL to generate the recombinant virus. The cells were observed every 12 h for the expression of fluorescence and harvested at 72 h post-transfection for propagation in eggs. These materials were then inoculated into the allantoic cavity of 10-day-old embryonated SPF chicken eggs to enable the efficient propagation of recovered recombinant viruses. Three successive passages in embryonated SFP chicken eggs were performed with a volume of 100 μl for each egg. All of the allantoic fluid from these eggs was harvested for further tests. The allantoic fluid harvested from each inoculated egg was tested for hemagglutination (HA) and hemagglutinin inhibition (HI) titers; for these tests, anti-NDV serum collected from NDV-immunized SPF chickens was used.

### Characterization of the rescued viruses

Viral RNA from the rescued viruses was extracted from fresh allantoic fluid following RT-PCR, and the full-length sequencing was performed as described above. The full-length genomic sequence was obtained from nine overlapping fragments. The viruses propagating in these eggs were genetically identified by comparison with the sequences of the parental virus.

Mean death time (MDT), intracerebral pathogenicity index (ICPI) and intravenous pathogenicity index (IVPI) were calculated to determine the virulence of rescued viruses, as described by the world organization for animal health (OIE) [36]. In each test, the parental virus 9a5b was utilized as the positive control. To determine the MDT value, the virus was serial 10-fold diluted, and then each dilution was inoculated into the allantoic cavities of five 9- to 11-day-old SFP embryonated eggs. Those inoculated eggs were incubated at 37 °C and checked every 12 h for 7 days. The MDT was the mean time in hours for the embryo death in the highest dilution at which all eggs died.

To determine the ICPI value, 0.05 ml of serial diluted virus was injected intracerebrally into each of ten one-day old SPF chickens. The birds were scored for disease symptoms and mortality (0 for normal, 1 for sick, and 2 for dead) every 24 h for 8 days. The ICPI is the mean score per bird per observation over the 8-day period.

To determine the IVPI value, viruses were inoculated intravenously into ten 6-week old SPF chickens. The birds are scored for disease symptoms and mortality (0 if normal, 1 if sick, 2 if paralyzed, and 3 if dead) over a period of 10 days. The IVPI value is the mean score per bird per observation.

Care and maintenance of all chickens were in accordance with the animal study protocol 11–08 of the Institutional Animal Care and Use Committee (IACUS) guidelines set by Shanghai Veterinary Research Institute, the Chinese Academy of Agricultural Sciences (CAAS).

### Growth kinetics of recovered virus and its parental virus

The propagation efficiency of recovered NDV and the parental virus were compared via a multiple-step growth cycle in DF-1 cells. DF-1 cells at 80 % confluency were transfected with each virus at a multiplicity of infection (MOI) of 0.01, and the supernatant was harvested at 2, 4, 6, 8, 12, 16, 24, 36, and 48 h post-infection. A 50 % tissue culture infection dose (TCID_50_) test was performed to quantify the live viruses secreted into the supernatant. Briefly, virus was serially diluted 10-fold and inoculated on DF-1 cells in 96-well plates. At 3 days post-infection, the lesions in the cells were observed under a microscope. Viral TCID_50_ titer in cell culture was calculated by the Reed and Muench method [37]. The experiment was performed in triplicate and a kinetic curve based on respective TCID_50_ was constructed for each strain.

## Abbreviations

APMV, avian paramyxovirus; CEFs, chicken embryo fibroblasts; F, fusion protein; GFP, green fluorescence protein; HA, hemagglutination; HI, hemagglutinin inhibition; HN, hemagglutinin-neuraminidase; ICPI, intracerebral pathogenicity index; L, large protein; M, matrix protein; MDT, mean death time; MOI, multiplicity of infection; NCR, non-coding region; ND, Newcastle disease; NDV, Newcastle disease virus; NP, nucleoprotein; OIE, the world organization for animal health; P, phosphoprotein; SPF, specific-pathogen-free; RACE, rapid amplification of cDNA ends; RT-PCR, reverse-transcription polymerase chain reaction; SPF, specific-pathogen-free; TCID50, 50 % tissue culture infection dose; UTR, untranslated region.

## Competing interests

The authors declare that they have no competing interests.

## Authors’ contributions

YY contributed for the construction of all the plasmids. XQ coordinated with the experiment design and drafted the manuscript. DX and HC carried out the construction of full-length NDV cDNA clone. YZ, LT and CM constructed the helper plasmids. NW performed the virus propagation in embryonated eggs. SY contributed manuscript editing. CD, XL and AQ made substantial contributions to the experiment design. All authors' have read and approved the final manuscript.
